# Beyond the lesion site: minocycline augments inflammation and anxiety-like behavior following SCI in rats through action on the gut microbiota

**DOI:** 10.1186/s12974-021-02123-0

**Published:** 2021-06-26

**Authors:** Emma K. A. Schmidt, Pamela J. F. Raposo, Abel Torres-Espin, Keith K. Fenrich, Karim Fouad

**Affiliations:** 1 Neuroscience and Mental Health Institute, University of Alberta, Edmonton, Alberta Canada; 2 Faculty of Rehabilitation Medicine, University of Alberta, 3-48 Corbett Hall, Edmonton, Alberta T6G 2G4 Canada; 3 Department of Physical Therapy, University of Alberta, Edmonton, Alberta Canada; 4 Department of Neurological Surgery, University of California San Francisco, Brain and Spinal Injury Center, San Francisco, CA USA

**Keywords:** Spinal cord injury, Minocycline, Inflammation, Microbiota

## Abstract

**Background:**

Minocycline is a clinically available synthetic tetracycline derivative with anti-inflammatory and antibiotic properties. The majority of studies show that minocycline can reduce tissue damage and improve functional recovery following central nervous system injuries, mainly attributed to the drug’s direct anti-inflammatory, anti-oxidative, and neuroprotective properties. Surprisingly the consequences of minocycline’s antibiotic (i.e., antibacterial) effects on the gut microbiota and systemic immune response after spinal cord injury have largely been ignored despite their links to changes in mental health and immune suppression.

**Methods:**

Here, we sought to determine minocycline’s effect on spinal cord injury-induced changes in the microbiota-immune axis using a cervical contusion injury in female Lewis rats. We investigated a group that received minocycline following spinal cord injury (immediately after injury for 7 days), an untreated spinal cord injury group, an untreated uninjured group, and an uninjured group that received minocycline. Plasma levels of cytokines/chemokines and fecal microbiota composition (using 16s rRNA sequencing) were monitored for 4 weeks following spinal cord injury as measures of the microbiota-immune axis. Additionally, motor recovery and anxiety-like behavior were assessed throughout the study, and microglial activation was analyzed immediately rostral to, caudal to, and at the lesion epicenter.

**Results:**

We found that minocycline had a profound acute effect on the microbiota diversity and composition, which was paralleled by the subsequent normalization of spinal cord injury-induced suppression of cytokines/chemokines. Importantly, gut dysbiosis following spinal cord injury has been linked to the development of anxiety-like behavior, which was also decreased by minocycline. Furthermore, although minocycline attenuated spinal cord injury-induced microglial activation, it did not affect the lesion size or promote measurable motor recovery.

**Conclusion:**

We show that minocycline’s microbiota effects precede its long-term effects on systemic cytokines and chemokines following spinal cord injury. These results provide an exciting new target of minocycline as a therapeutic for central nervous system diseases and injuries.

**Supplementary Information:**

The online version contains supplementary material available at 10.1186/s12974-021-02123-0.

## Background

Spinal cord injury (SCI) causes permanent loss of function and disability below the level of injury. Additional effects of SCI include autonomic dysreflexia, immune suppression, intestinal dysbiosis, and an increased risk of mental health disorders [1–10]. Such system-wide consequences of SCI are not only detrimental to the quality of life but can also lead to increased mortality rate [11–14]. Considering these multiple aspects of recovery following SCI, treatments with effects beyond the lesion site (e.g., on systemic inflammation, gut dysbiosis, and mental health) are therefore vital to promote comprehensive treatment options.

Minocycline is a synthetic tetracycline derivative that has been shown to have anti-inflammatory, anti-oxidative, and direct neuroprotective effects after SCI [15–21]. These positive preclinical results, coupled with minocycline’s long safety record in humans [22], resulted in a phase II placebo-controlled randomized clinical trial to test the therapeutic effects of minocycline treatment for acute SCI [23]. The results from this trial showed modest, though not statistically significant, effects on motor recovery in people with SCI that received minocycline [23]. Beneficial neuroprotective effects of minocycline have also been shown in amyotrophic lateral sclerosis, stroke, multiple sclerosis, and Parkinson’s disease [24–27]. The pharmacological effects of minocycline have primarily been attributed to the modulation of neuroinflammation [28, 29]. However, various studies have failed to reproduce the neuroprotective effects of minocycline treatment following SCI, Parkinson’s disease, and Huntington’s disease [30–32]. Another study reported no significant functional improvement between the minocycline and placebo groups in individuals with acute traumatic brain injury [33], further highlighting the contradicting findings on the efficacy of minocycline treatment.

Minocycline is also a broad-spectrum antibiotic, modulating the composition of the intestinal microbiota [34, 35]. Recently, an imbalanced intestinal microbiota composition (dysbiosis) has been linked to impaired functional recovery and increased anxiety-like behavior following SCI [9, 10]. Bidirectional communication between the microbes that colonize the gastrointestinal tract and the central nervous system can have a profound impact on disease progression and likely involves interactions with the host immune system [36–38]. Although the local tissue response to minocycline treatment for SCI has been well characterized, minocycline’s (and antibiotics in general) impact on the microbiota-immune axis following SCI is unknown. This is particularly relevant since 93.2% of SCI patients receive antibiotic treatment in the first week after injury [39–41]. Such systemic effects of minocycline beyond the lesion site may help explain the contradicting evidence of minocycline’s efficacy as a treatment for SCI.

The aim of the present study was to elucidate multiple system-wide changes induced by minocycline treatment in a rodent model of cervical SCI. Four groups of rats were tested: uninjured, uninjured + minocycline, SCI, and SCI + minocycline. We show, for the first time, that minocycline treatment for SCI has a profound acute effect on the fecal microbiota diversity and composition and subsequently prevents SCI-induced suppression of cytokines/chemokines and attenuates anxiety-like behaviors.

## Materials and methods

### Animals

All animal use was approved by the Animal Care and use Committee for Health Sciences at the University of Alberta. Adult female Lewis rats (Charles River, *n* = 40, 8–9 weeks and 180–220 g upon arrival) were group-housed with 5 rats per cage (experimental groups housed separately). Female rats were chosen in order to compare the findings with our previous experiments on microbiota changes following SCI [10]. Rats were kept on a 12-h light/dark cycle (lights on at 08:00), and they received ad libitum access to standard rat chow and water. Behavioral testing and analyses were performed by an experimenter blind to the experimental groups. Rats were divided into four groups; four rats were excluded (one died after surgery, one had a lesion size of 0%, one had a lesion size greater than 50%, and one was a multidimensional outlier for the plasma and microbiota analysis) for a total of 36 rats: uninjured *n* = 10, uninjured + minocycline *n* = 10, SCI *n* = 8, and SCI + minocycline *n* = 8.

### Drug administration

A 50-mg/kg minocycline hydrochloride (Sigma Aldrich) was dissolved in sterile water and administered via oral gavage daily for 7 days beginning 2 h after SCI. Rats that did not receive minocycline were gavaged with 0.5 ml sterile water daily for 7 days beginning 2 h after SCI.

### Spinal cord injury

Surgeries were performed similarly to previously described [10]. Under isoflurane anesthesia (5% induction; 2.5% maintenance, supplied with a 50:50 air/oxygen mixture), the dorsal neck was shaved and disinfected with 10% chlorhexidine digluconate (Sigma-Aldrich). A 125 kdyn unilateral contusion was performed 1.25 mm right of the midline at an angle of 15° (pointed towards the midline) at C5 using an Infinite Horizons impactor (Precision Systems & Instrumentation). The muscles were sutured with absorbable sutures, and the skin was closed with 9 mm stainless steel clips. Buprenorphine (0.03 mg/kg, WDDC) was administered subcutaneously as a post-operative analgesic and again 8–12 h later. Animals received 4 ml of sterile 0.9% saline solution (subcutaneous) for hydration immediately after surgery. The bladders were manually expressed when necessary (evidence of wet abdomen and full bladder) until the animal re-established voluntary control of micturition.

### Behavioral testing

Tests were performed during the light cycle by an experimenter blind to the group conditions. All testing apparatuses were cleaned with unscented soap and water and dried between each animal. Baseline testing was performed on the open field and cylinder tests. The elevated plus maze and light-dark box tests were used only once to avoid one-trial tolerance [43].

### Open field

To assess general locomotor activity, rats were placed in the center of an open-field arena (100 × 80 × 30 cm) for 5 min while video recorded from above [44]. Offline video analysis of the distance traveled was performed using customized tracking software (https://github.com/cdoolin/rat-apps).

### Elevated plus maze

To evaluate anxiety-like behavior, rats were placed in the junction of two open arms and two closed arms (each arm is 50-cm long and 10-cm wide) elevated 65 cm above the ground while being video recorded from above for 10 min [45]. Offline video analysis was performed using customized motion tracking software (https://github.com/cdoolin/rat-apps) to analyze the time spent in the open arms and total distance traveled. Entries into the open and closed arms of the elevated plus maze (EPM) were counted when all 4 paws were located in the arm.

### Cylinder

To evaluate forepaw asymmetry, rats were video recorded while they explored the walls of a clear plexiglass cylinder (21 cm diameter × 25 cm height) for 5 min [46]. The numbers of left and right paw placements were recorded and expressed as a percentage of ipsilesional paw placements.

### Light-dark box

To assess anxiety-like behavior, rats were placed in the dark compartment of the light-dark box (LDB) (dark compartment 0 lux, light compartment 100 lux) and video recorded from above for 10 min [47]. Total distance traveled and the integer number of entries (considered when all 4 paws enter the light box) into the light compartment were recorded using custom software.

### Sucrose preference test

The sucrose preference test was used to evaluate anhedonia, a symptom of depressive-like behavior [48]. Rats received access to 2 water bottles in their home cage: one with a 1% sucrose solution and the other with regular drinking water (rats were not acclimatized to the sucrose water prior to the testing period). The percentage of sucrose water consumed over 2 h was recorded during the dark cycle when the rats were more active. The location of the water bottles was switched after 1 h to control for side preference.

### Fecal collection

To collect fresh fecal matter for 16s rRNA analysis, rats were placed in individual sterile cages at the beginning of the dark cycle as previously described [10]. Fecal pellets were individually collected in sterile microcentrifuge tubes and immediately frozen at − 80 °C until further processing.

### Blood collection

To collect blood for subsequent plasma analysis, animals were gently restrained and the area over the tarsal joint was shaved. Blood was always collected at the beginning of the light cycle. The saphenous vein was punctured using a sterile needle, and blood was collected into a microvette CB300 capillary tube (Sarstedt Inc., Nümbrecht, Germany) and kept on ice. Blood samples were then centrifuged for 5 min at 3000 rpm at 4 °C; the supernatant plasma was aliquoted into sterile microcentrifuge tubes and frozen at − 80 °C until further processing.

### Cytokine analysis

Plasma samples diluted 2-fold and run on the Rat Cytokine 27-Plex and Rat Stress Hormone 2-Plex discovery assays (Eve Technologies, Calgary, Canada) that measured: Eotaxin, EGF, Fractalkine, IFN-gamma, IL-1a, IL-1b, IL-2, IL-4, IL-5, IL-6, IL-10, IL-12(p70), IL-13, IL-17A, IL-18, IP-10, GRO/KC, TNF-alpha, G-CSF, GM-CSF, MCP-1, Leptin, LIX, MIP-1alpha, MIP-2, RANTES, VEGF, corticosterone, and melatonin. All plasma analytes were normalized to baseline (before injury) values for analysis (fold change calculated as the analyte value subtracted by the baseline value, then divided by the baseline value).

### Multivariate analysis of plasma analytes

The fold change with respect to the baseline was used to normalize the expression of each analyte to its respective pre-injury levels. A multivariate analysis of variance (MANOVA) considering repeated measures was used to test the hypothesis that the mean vector of the groups and their interaction over time were different in the plasma analytes variable space. The Wilks test was used to determine significance. To study the temporal patterns, multiple factor analysis [49] was computed using the *FactoMiner* R package [50] to extract the multivariate scores of each animal in the plasma analyte landscape and the loadings of each analyte at different time points. Each time point constituted a group of variables of plasma analytes using the fold increase with respect to baseline. We considered the first 2 dimensions for further analysis, explaining 24.3% and 12.4% of the total variance by dimension 1 and dimension 2, respectively. Linear mixed model (LMM) was fitted for dimension 1 for statistical inference considering group, time, and their interaction as fixed effect terms and the animal as a random effect using the *lme4* and *lmerTest* R packages [51, 52].

### 16s rRNA analysis

Frozen fecal samples were shipped on dry ice to Microbiome Insights Inc. (Vancouver, Canada) for sequencing and bioinformatics. 16Sv4 amplicons were generated from the fecal samples, and MiSeq-generated Fastq files were quality filtered and clustered into 97% similarity operational taxonomic units (OTUs) using the mothur software package (v. 1.39.5) (Schloss et al. 2009). The resulting dataset consisted of 184,614 OTUs with an average of 33,185 reads per sample. OTUs were removed if their mean abundance in controls reached or exceeded 25% of their mean abundance in specimens. Alpha diversity was estimated with the Shannon index on raw OTU abundance tables after contaminants were filtered out. Putative contaminants were described as OTUs whose mean abundance in controls was equal to or greater than 25% of their mean abundance in the sample specimens.

### Multivariate analysis of microbiota composition

OTU data was analyzed using R [53] through Rstudio [54]. Abundance tables for each time point were normalized with respect to each animal’s baseline by *x*_*i*, *t*_/(*x*_*i*, *t*_ + *x*_*i*, *bl*_), where *i* is the animal, *t* the time point, and *bl* represents the value at baseline. Unsupervised ordination of the normalized abundance table was conducted (for each of the aggregated taxonomic levels: species, genus, family, class, order, and phylum) blinded to the experimental condition by non-metric multidimensional scaling of the Bray-Curtis distance between animals using the *vegan* R package [55] with a maximal of 20 iterations and keeping the first 5 dimensions. A permutation multivariate analysis of variance (PERMANOVA) was computed using the *vegan* package over 999 permutations of the Bray-Curtis dissimilarity matrix to test the hypothesis of whether the centroids of the multivariate space were different by the terms of group, time, and their interaction. Pairwise comparisons were performed using the *pairwiseAdonis* R package [56], adjusting *p* values using Bonferroni’s correction.

### PICRUSt2 analysis

The PICRUSt2 (Phylogenetic Investigation of Communities by Reconstruction of Unobserved States) software was used following the developer’s instructions to predict the functional abundances based on the 16s rRNA gene sequences [57, 58]. The relative abundance was calculated by dividing the abundance of each pathway by the total abundance of all pathways per sample. The top 10% most abundant pathways were used for analysis and presented as a fold change from baseline values.

### Perfusion and tissue cutting

Animals were euthanized 5 weeks post-SCI with a lethal dose of sodium pentobarbital (240 mg/kg). Rats were transcardially perfused with saline containing 0.02 g heparin/l followed by 4% paraformaldehyde in 0.1 M phosphate-buffered saline (PBS) with 5% sucrose. The spinal cords were extracted and post-fixed overnight in 4% PFA at 4 °C followed by 30% sucrose for 5 days. The cervical spinal cord containing C3–C7 (1-cm block) was embedded in O.C.T. (Sakura Finetek, USA) mounted onto filter paper and frozen in 2-methylbutane (− 40 °C). The serial cross-sections of the spinal blocks were cut at a thickness of 25 μm on a NX70 cryostat (Fisher Scientific), staggered across eight sets of slides, and stored at − 20 °C until further processing.

### Lesion analysis

Frozen sections were thawed for 1 h at 37 °C and rehydrated in TBS (2 × 10 min). Slides were placed in 0.5% cresyl violet acetate solution for 3 min and dehydrated following concentrations of 50% and 70% and absolute ethanol for 2 min each. Slides were cleared with histological xylene solution (2 × 2 min) and mounted using Permount (Fisher Scientific) mounting medium. The total rostral-caudal extension of the lesion was imaged using a light microscope (Leica DM6000B, camera Leica DFC350 FX) and quantified using the ImageJ software (National Institute of Health, USA). The lesioned area was defined as the lesion cavity and surrounding the scar tissue. For each spinal cord cross-section, the lesioned area was divided by the total area of the cross-section and expressed as a percentage.

### Immunohistochemistry

Frozen sections were thawed at 37 °C for 1 h and rehydrated in PBS for 2 × 10 min followed by PBS with 0.3% Triton™ X-100 (PBS-T) for 10 min. Five percent normal donkey serum in PBS-T was applied as a blocking buffer for 1 h at room temperature. The sections were then incubated overnight at 4 °C in an anti-IBA1 rabbit (1:500, Wako) antibody with a blocking buffer. The next morning, the sections were washed with PBS-T (3 × 10 min) and incubated with donkey anti-rabbit AF488-conjugated (1:500, Life Technologies) antibody in a blocking buffer for 2 h at room temperature. The sections were then rinsed 3 × 10 min in PBS and cover-slipped with Fluoromount-G (Southern-Biotech).

### Image analysis

Images were captured using an epifluorescence microscope (Leica DM6000B, camera Leica DFC350 FX) and analyzed using ImageJ. Images were acquired at × 5 magnification to visualize the entire spinal cord cross-section 0.25 cm rostral to the lesion, at the lesion epicenter, and 0.25 cm caudal to the lesion. IBA1 fluorescent optical density was quantified and expressed as a percentage area of positive staining using thresholding. To assess microglial morphology, a × 40 magnification image was taken of the ventral horn of the gray matter on both the ipsilesional and contralesional side rostral (0.25 cm), at, and caudal (0.25 cm) to the lesion. Three representative cells per image were chosen, and the process length and number of endpoints per cell were measured using the ImageJ plugin NeurphologyJ [59].

### Statistical analysis

Behavioral, tissue, and plasma statistical analysis was performed using GraphPad Prism 8 (San Diego, CA). A 5% or less alpha value was considered significant. Time-course data was analyzed using a repeated-measure two-way ANOVA with the Geisser-Greenhouse correction followed by Tukey’s multiple comparison post hoc test, with individual variances computed for each comparison. For data with only one time point, a one-way ANOVA was used followed by Fisher’s LSD test. All summary values in the text represent mean ± standard deviation if not otherwise stated.

## Results

### Minocycline treatment did not affect lesion size

**Fig. 1:**
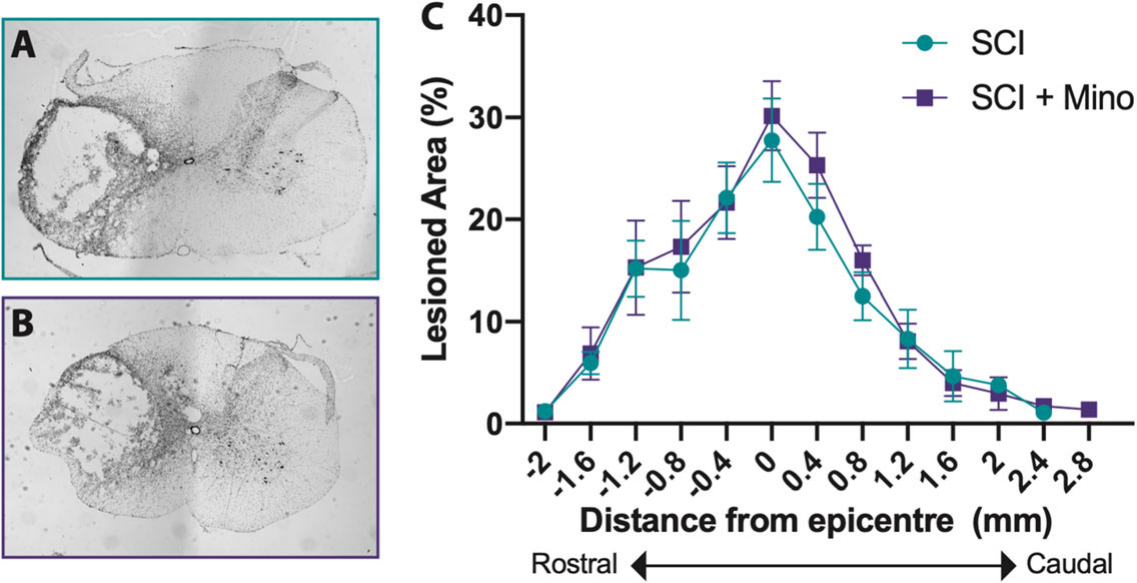
Minocycline treatment had no effect on lesion size. Representative images of the maximum lesioned area for the SCI group (**a**) and SCI + minocycline group (**b**). **c** The rostral (negative numbers) to caudal (positive numbers) extension of the lesion was quantified and expressed as the percentage of the lesioned area for each coronal section. Error bars represent the standard error of the mean

To determine whether minocycline treatment reduced lesion size following SCI, the rostral to the caudal extension of the lesioned area in the coronal plane was analyzed 5 weeks after injury. There was no difference between minocycline treated or untreated rats in the size (SCI 27.74% ± 11.48%; SCI + minocycline 31.38% ± 10.07%) or extension (SCI 3.25 mm ± 0.89 mm; SCI + minocycline 3.75 mm ± 0.56 mm) of the lesion (Fig. 1a–c).

### Minocycline altered microglial density and morphology

**Fig. 2:**
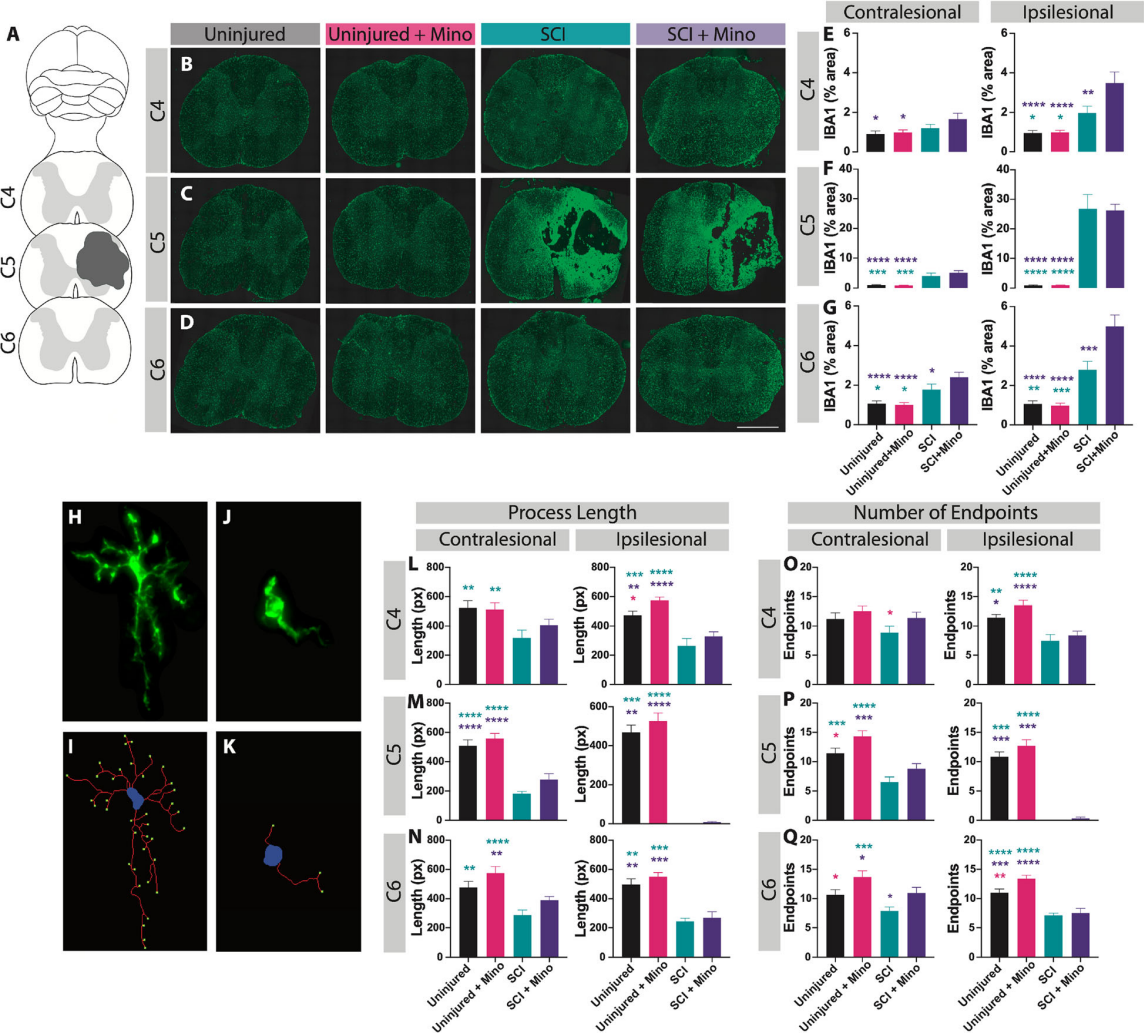
Minocycline treatment altered the microglial density and morphology around the lesion site. **a** IBA1 immunohistochemical marker was used to visualize the microglia in the cervical spinal cord at C4, at the maximum lesion site (C5), and caudal to the lesion site at C6. Representative spinal cord images are shown from each group at C4 (**b**), C5 (**c**), and C6 (**d**). Quantification of the area of IBA+ staining is shown at C4 (**e**), C5 (**f**), and C6 (**g**) on the contralesional (left graphs) and ipsilesional (right graphs) spinal cord. Microglial morphology in the ventral gray matter was assessed by quantifying the length and number of endpoints per cell. **h** Image of a ramified microglia and **i** the automated analysis shows the soma in blue, processes in red, and the endpoints in green. **j** Image of an activated microglia and **k** the corresponding output of the analysis. Quantification of the average process length per cell is shown at C4 (**l**), C5 (**m**), and C6 (**n**) on the contralesional (left graphs) and ipsilesional (right graphs) spinal cord. Quantification of the average number of process endpoints per cell is shown at C4 (**o**), C5 (**p**), and C6 (**q**) on the contralesional (left graphs) and ipsilesional (right graphs) spinal cord. Error bars represent the standard error of the mean. Colored asterisks represent which group is significantly different. Scale bar represents 1 mm. *p < 0.05, **p < 0.01. ***p < 0.001, ****p < 0.0001

The density and distribution of the microglia marker, IBA1, were analyzed around the lesion site at C4, C5 (at the site of maximal lesion extent), and C6 (Fig. 2a–d). It is well characterized that SCI results in the activation of microglia [60], which was confirmed by the increased area of IBA1 staining in all SCI rats relative to uninjured groups. Minocycline treatment following SCI resulted in an increased area of IBA1 immunoreactivity rostral to the injury at C4 (ipsilesional: *p* = 0.003) and caudal to the injury at C6 (contralesional: *p* = 0.048, ipsilesional: *p* = 0.0001) compared to untreated SCI rats (Fig. 2e–g). However, there was no significant difference in the IBA1 immunoreactivity at the lesion epicenter between the SCI groups (Fig. 2f). Lastly, there was no effect of minocycline treatment between the uninjured groups in IBA1 staining at any location measured.

Microglial morphology was further assessed in the ventral gray matter by quantifying the length of the microglial processes and the number of endpoints per cell (Fig. 2h–k). Increased process length and endpoints suggest a ramified microglial morphology, whereas a reduction in the length and number of endpoints indicates a more activated state [61, 62]. SCI resulted in a significant increase in the activation of microglia characterized by decreased process length (Fig. 2l–n) and reduced number of endpoints (Fig. 2o–q) per cell. Focusing on the effect of the treatment, rats that received minocycline displayed a general increase in the process length, which was significant between the uninjured groups at C4 (Fig. 2l, ipsilesional: *p* = 0.032). Similarly, minocycline treatment resulted in an overall increase in the number of endpoints per cell in both uninjured and injured groups. This was significant between the uninjured groups at C5 (ipsilesional: *p* = 0.027) and C6 (ipsilesional: *p* = 0.008, contralesional: *p* = 0.025) and between the SCI groups at C6 (ipsilesional: *p* = 0.035). In summary, 7 days of minocycline treatment induced a more ramified spinal microglial morphology in both uninjured and SCI rats relative to the untreated control groups measured 28 days after the offset of the treatment.

### Minocycline promoted affective but not motor recovery following SCI

**Fig. 3:**
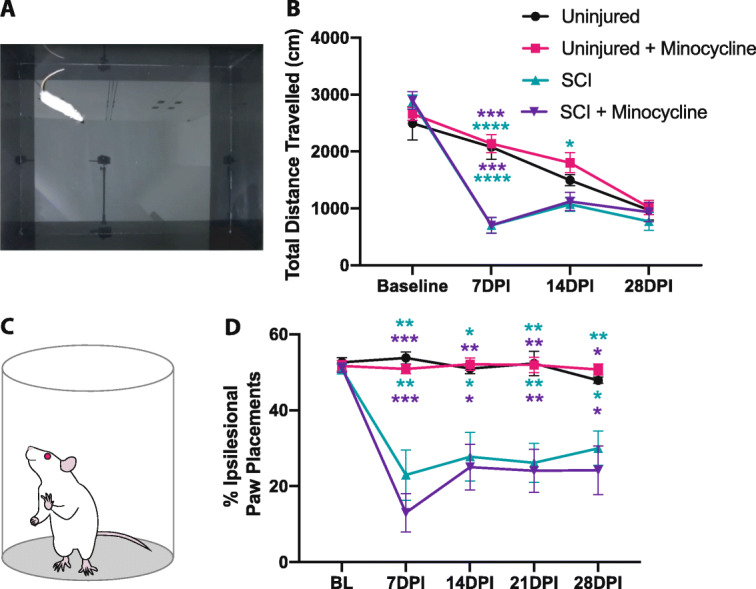
Minocycline treatment did not affect motor recovery in the open field or cylinder task. **a** Image showing a rat in the center of the open field apparatus. **b** Both SCI and SCI + minocycline groups traveled significantly less distance than uninjured rats in the open field at 7 days post-SCI. **c** The cylinder test was used to assess forepaw use asymmetry. **d** SCI resulted in decreased use of the ipsilesional paw compared to uninjured rats. Error bars represent the standard error of the mean. *p < 0.05, **p < 0.01, ***p < 0.001, ****p < 0.0001. Top asterisks represent the uninjured + minocycline groups vs. SCI (green) and SCI + minocycline (purple). Bottom asterisks represent uninjured vs. SCI (green) and SCI + minocycline (purple)

Rat behavior was assessed at baseline (prior to SCI) and for 4 weeks following injury. Both the SCI and SCI + minocycline groups had a drop in body weight following injuries which returned to uninjured values by 4 weeks (time × group effect *p* < 0.0001, time effect *p* < 0.0001, group effect *p* = 0.006). Uninjured rats that received minocycline consistently had the highest body weight (Additional file 1). At 7 days post-injury, both the SCI groups traveled significantly less distance in the open field compared to uninjured rats (Fig. 3a, b; time × group effect *p* < 0.0001, time effect *p* < 0.0001, group effect *p* = 0.001). By 14 days post-injury, untreated SCI rats traveled significantly less distance than uninjured + minocycline animals. By 28 days post-SCI, all groups regardless of injury had declined in the overall distance traveled in the open field (Fig. 3a, b). SCI resulted in a significantly reduced use of the ipsilesional forepaw in the cylinder test, with no effect of minocycline treatment (time × group effect *p* < 0.0001, time effect *p* < 0.0001, group effect *p* < 0.0001) (Fig. 3c, d).

**Fig. 4:**
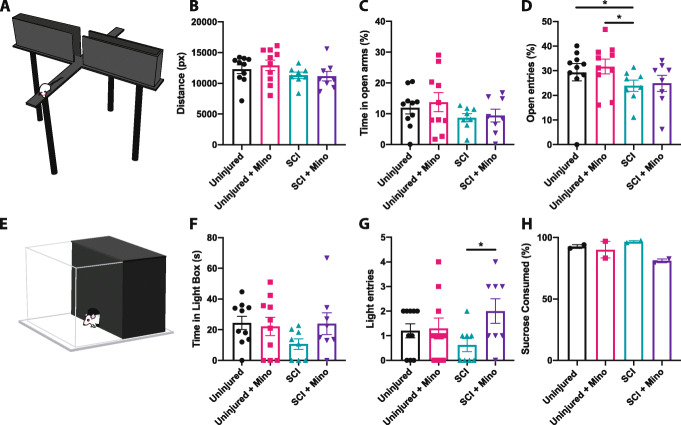
Minocycline treatment attenuated spinal cord injury-induced anxiety-like behavior. **a** Rat in the open arm of the elevated plus maze. **b** The total distance traveled in the maze. **c** The percent time in the open arms was calculated as a percentage of the time spent in the open arms divided by the total time spent in the maze. **d** The percent of open arm entries was calculated as the percentage of the number of open arm entries divided by the total open and closed arm entries. **e** Schematic showing a rat entering the light component of the light-dark box. **f** The amount of time spent in the light component and **g** the number of entries made into the light component of the light-dark box. **h** The percent of sucrose water consumed where each data point represents a cage. Error bars represent the standard error of the mean. *p < 0.05

A single testing session 3 weeks post-SCI was used for the EPM and LDB to avoid one-trial tolerance [43, 63]. This time point was chosen to minimize the interference of locomotor deficits following acute SCI as we have previously reported similar distances traveled in the EPM between the uninjured and SCI groups by 3 weeks post-injury [10]. Similarly, there was no difference between all four experimental groups in the total distance traveled in the EPM (Fig. 4a, b). Comparable to our previous study reporting SCI-induced anxiety-like behavior in the EPM [10], untreated SCI rats spent less percent time in the open arms and made significantly fewer open arm entries than both uninjured groups (uninjured vs. SCI *p* = 0.047, uninjured + minocycline vs. SCI *p* = 0.028) (Fig. 4c, d). Paralleling the SCI-induced anxiety-like behavior observed in the EPM, untreated SCI rats spent the least amount of time in and entries into the light compartment of the LDB (Fig. 4e–g). SCI + minocycline rats made significantly more entries into the light compartment compared to untreated SCI rats, indicating reduced anxiety-like behavior (*p* = 0.022). Anhedonic behavior was assessed in the sucrose preference test 7 days post-injury (at the offset of minocycline treatment). SCI + minocycline rats consumed the least amount of sucrose water; however, this did not reach statistical significance (Fig. 4h). Taken together, results from these behavioral tests suggest that minocycline treatment had a long-term (i.e., at least 2 weeks after the offset of treatment) attenuation of anxiety-like behavior in the LDB but did not promote motor recovery following SCI.

### Minocycline prevented SCI-induced suppression of inflammatory cytokines/chemokines in the plasma

**Fig. 5:**
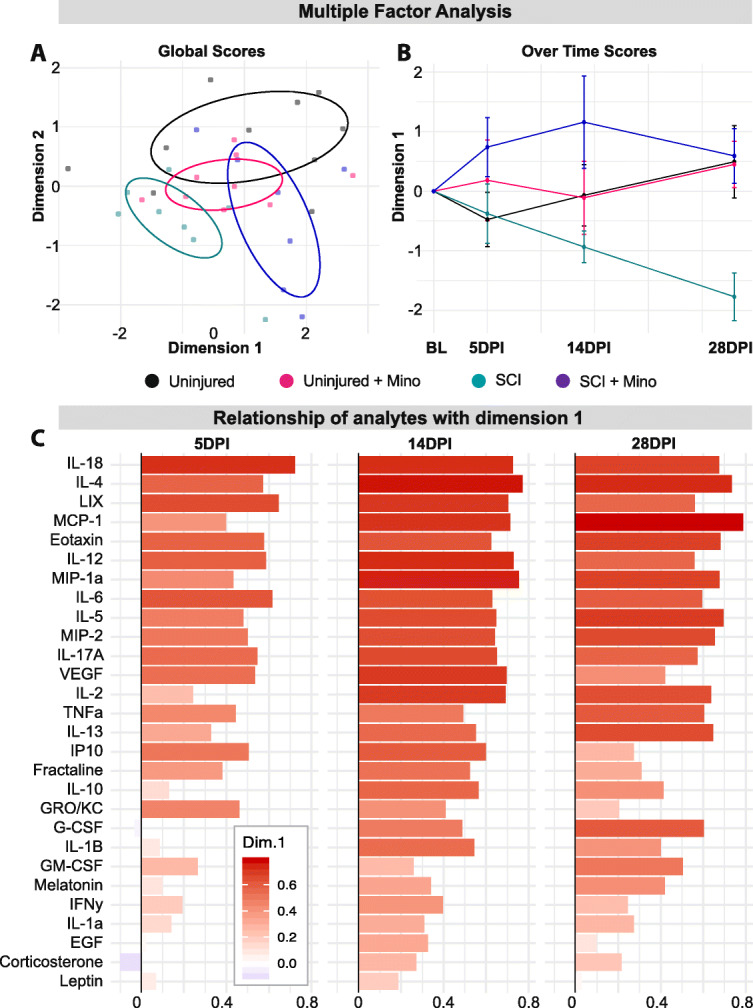
Minocycline treatment prevented spinal cord injury-induced long-term changes in plasma cytokines. **a** Multiple factor analysis of plasma analytes (measured with respect to the baseline values) shows the relationship of each subject with the multidimensional factors 1 and 2 across all time points. **b** Scores in multidimensional factor 1 are shown for each group over time. **c** The importance of each plasma analyte to multidimensional factor 1 is shown over time using their loadings. Ellipses in **a** represent the 50% bivariate distribution. Error bars represent the standard error of the mean

**Fig. 6:**
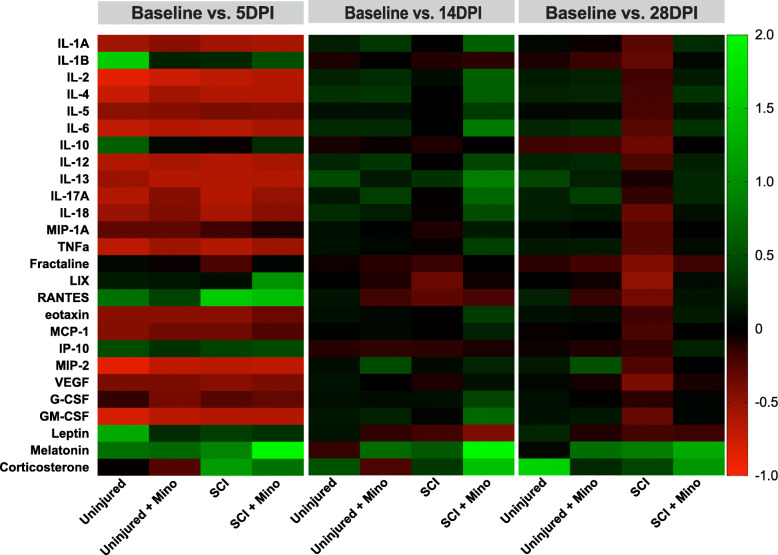
Minocycline prevents spinal cord injury-induced suppression of plasma cytokines and chemokines. Heatmap showing the relative change of plasma analytes at 5, 14, and 28 days post-injury. Positive numbers reflect an increase and negative numbers reflect a decrease from baseline values

A total of 29 plasma analytes (cytokines, chemokines, and hormones) were measured at 5, 14, and 28 days post-SCI and expressed as a fold change from baseline values. Multivariate analysis of variance resulted in a significant group by time interaction (MANOVA group x time *p* = 0.005), indicative of differences across the groups and time in the overall profile of plasma analytes. Multiple factor analysis was used to study the multidimensional relationship between plasma analytes, group, and time. Global scores show differences between the groups across time, particularly in dimension 1 (Fig. 5a). When looking at the dimension 1 scores at each time point measured, there is a deviation of untreated SCI rats from the uninjured groups by 28 days post-injury (Fig. 5b). Minocycline treatment prevented this SCI-induced long-term change in the plasma analyte composition. Looking at the relationship of the individual plasma analytes with dimension 1, all plasma markers (with the exception of G-CSF and corticosterone) moved towards the same positive direction (Fig. 5c). Given that the SCI + minocycline and uninjured groups are mostly positive in dimension 1, this indicates that there is a correlation between these groups and the changes in plasma analytes, which is opposite in direction to the untreated SCI group. Looking at individual plasma analytes, there was a substantial downregulation in the majority of analytes at 5 days post-injury in all groups (Fig. 6). By 14 days post-injury, there were minimal differences between the treatment groups in levels of plasma analytes (Fig. 6). By 28 days post-injury, SCI induced a significant suppression of the majority of plasma cytokines and chemokines, which was normalized with minocycline treatment (Fig. 6). Additional file 2 shows the univariate adjusted *p* values for the individual plasma analytes that were significantly different between the groups at each time point with respect to baseline.

### Minocycline treatment altered the diversity, composition, and predicted function of the fecal microbiota

**Fig. 7:**
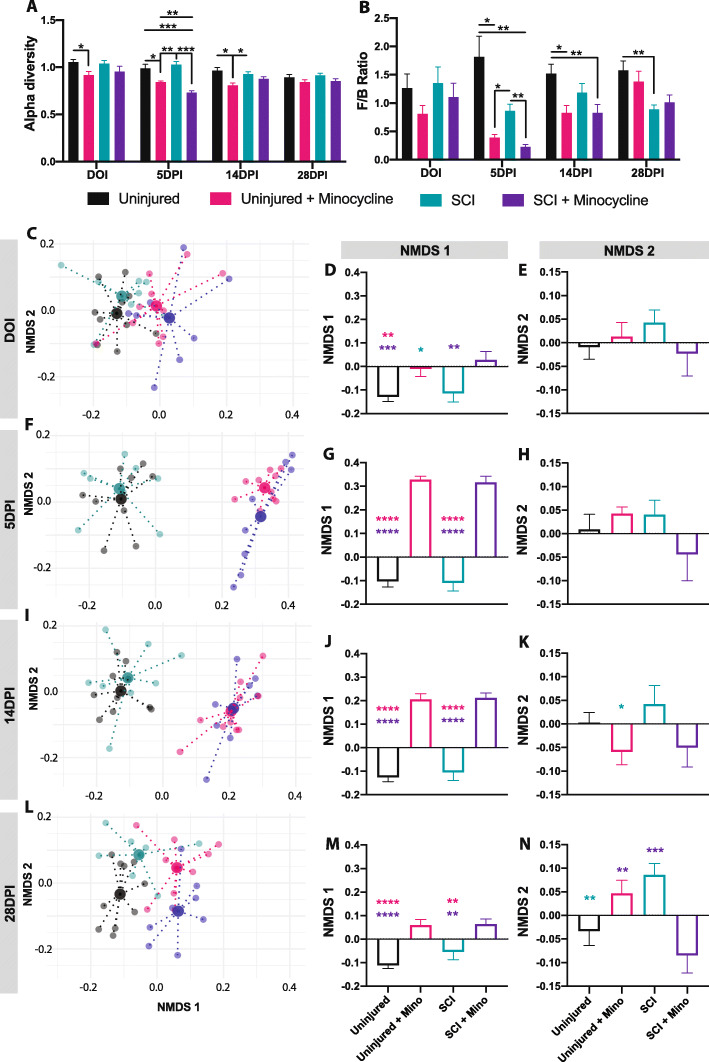
Minocycline treatment altered the gut microbiota composition. **a** The Shannon index of alpha diversity and **b** the Firmicutes/Bacteroidetes ratio are shown over time for each treatment group. **c**–**n** Non-metric multidimensional scaling (NMDS) was used to visualize the overall microbiota composition at the phylum level. **c** 2D plot of the NMDS first 2 components shows the centroid of each group (large points) and each individual rat (small points) on the day of injury. Individual NMDS 1 scores (**d**) and NMDS 2 scores (**e**) are shown for each group on the day of injury. Similar plots are shown for 5 days post-injury (**f**–**h**), 14 days post-injury (**i**–**k**), and 28 days post-injury (**l**–**n**). All data is normalized to baseline values. Error bars represent the standard error of the mean. Colored asterisks represent which group is significantly different. *p < 0.05, **p < 0.01, ***p < 0.001

To determine the spectrum of microbiota changes induced by both SCI and minocycline, fecal samples were collected and 16s rRNA gene sequencing was performed at baseline, on the day of injury (DOI), and 5, 14, and 28 days post-injury (DPI) and expressed relative to baseline values. Minocycline treatment resulted in a significant decrease in the Shannon index of alpha diversity, confirming minocycline’s antibiotic effects (Fig. 7a). This reduction in bacterial diversity lasted longer in uninjured + minocycline rats (reduced alpha diversity on the first day of treatment, 5 and up to 14 days) and was shorter but more severe in SCI + minocycline rats (significantly decreased relative to all other groups at 5 days post-injury) (time × group effect *p* < 0.0001, time effect *p* < 0.0001, group effect *p* < 0.0001). The ratio of Firmicutes to Bacteroidetes (the two major bacterial phyla) was differentially affected by minocycline treatment and SCI (Fig. 7b). Minocycline treatment resulted in a transient but significant decrease in the Firmicutes/Bacteroidetes ratio that lasted up to 14 days post-SCI compared to untreated uninjured rats. SCI alone (without minocycline treatment) also decreased the Firmicutes/Bacteroidetes ratio relative to untreated uninjured rats, which reached statistical significance by 28 days post-injury (time × group effect *p* < 0.0001, time effect *p* = 0.004, group effect *p* = 0.002).

Non-metric multidimensional scaling (NMDS) was used to extract the landscape space of the microbiota composition at each taxonomic level. Focusing on the phylum level, minocycline resulted in a significant alteration of the microbiota composition beginning on the first day of treatment (on the day of injury) regardless of whether the animals received a SCI or were uninjured (Fig. 7c–e) (NMDS1 *p* = 0.0016). The difference between the minocycline treated and untreated groups is seen primarily in NMDS component 1, which was maximal at 5 (Fig. 7f–h) and 14 days (Fig. 7i–k) post-injury. These differences were reduced but still significant by 28 days post-injury (21 days after the offset of minocycline treatment) (Fig. 7l–n) (5 DPI *p* < 0.0001; 14 DPI *p* < 0.0001; 28 DPI *p* < 0.0001). Significant differences between the groups in NMDS component 2 emerged beginning at 14 days and was maximal by 28 days post-injury, when the uninjured and SCI + minocycline groups moved in the opposite direction to the uninjured + minocycline and SCI groups (Fig. 7n) (NMDS2 *p* = 0.002). NMDS plots for all other taxonomic levels can be found in the additional files 3–7. Looking at the family, class, and order taxonomic levels, untreated SCI rats deviated from all other groups at 28 days in NMDS component 2. Although there was a significant effect of SCI at 28 days post-injury in NMDS component 2 at multiple taxonomic levels, minocycline treatment accounted for the majority of changes in the microbiota composition regardless of injury. A similar trend is seen in the functional gene profile of the microbiota composition (Additional file 8). Looking at the top 10% most relatively abundant genetic pathways, the majority of differences were observed at 5 days post-injury between minocycline treated and untreated groups regardless of injury, suggesting that minocycline treatment also had a significant acute effect on the microbiota functional profile (Additional file 9). Together, these data indicate that minocycline has a significant acute effect on the microbiota diversity, composition, and function, which preceded minocycline’s effects on plasma cytokines and chemokines.

## Discussion

Minocycline has been widely studied for its direct anti-inflammatory and neuroprotective properties for central nervous system diseases and injuries including amyotrophic lateral sclerosis, stroke, multiple sclerosis, Parkinson’s disease and SCI [15, 16, 24–27, 63]. However, minocycline’s impact on the intestinal microbiota and systemic immune response following SCI has not yet been investigated. Using a comprehensive analysis of plasma inflammatory analytes and fecal microbiota, we show for the first time that minocycline treatment has a profound acute effect on the microbiota composition followed by the prevention of SCI-induced suppression of inflammatory cytokines/chemokines. Although minocycline did not reduce lesion size or improve motor recovery, it did have a modest anxiolytic effect after SCI.

In addition to being a broad spectrum antibiotic, minocycline is highly lipid soluble and can pass through the blood-brain barrier to produce a variety of anti-inflammatory, anti-oxidative and neuroprotective effects [16, 18, 28]. Minocycline has been shown to inhibit caspase-1, caspase-3 and microglial activation, protect neurons from oxidative stress and free radicals, prevent glutamate-induced apoptosis, and protect blood-brain barrier integrity [14, 16]. These promising preclinical results prompted a phase II placebo-controlled randomized clinical trial of minocycline to treat acute SCI [23]. Although recognized for its lack of adverse side effects, there was no statistically significant effect of minocycline efficacy for motor recovery after SCI [23]. Furthermore, a pivotal animal study reporting neuroprotective benefits of minocycline for cervical SCI was unable to be replicated in a follow up study [64, 65]. Another study found no behavioral or histological benefits of minocycline treatment for cervical contusion in rats [32]. Contradicting results of minocycline treatment have also been reported in stroke, Parkinson’s and Huntington’s diseases [30, 31]. In line with these studies, we found no beneficial effect of minocycline treatment on lesion size or motor recovery after SCI. However, the utilized motor tests in the present study may not have been sensitive enough to detect changes in fine motor skills. Curiously, SCI rats treated with minocycline displayed an increased density of IBA1 immunoreactivity above and below the injury site, which has not been reported previously. This increase in microglia density was not observed in minocycline treated uninjured rats, suggesting that minocycline treatment has differing effects on microglial cells depending on the local tissue environment. Although IBA1 is upregulated upon activation of microglia [66], we showed that the observed amplified area of IBA1+ cells in the SCI + minocycline group was not due to a morphological activated state of microglia. This was determined by the increased complexity of IBA1+ cells (i.e., increased process length and number of endpoints), indicating a ramified (less activated) microglial phenotype in rats that received minocycline, which has been previously reported [17, 18, 67]. The increased density of IBA1 immunoreactivity in SCI + minocycline rats may otherwise be due to the observed increase in length and number of microglial endpoints or a general increase in the number of microglial cells.

Many of the anti-inflammatory properties of minocycline have also been studied for their beneficial effects on depression and anxiety [29, 67, 68]. For example, minocycline can block lipopolysaccharide-stimulated inflammatory cytokine secretion, sickness behavior and anhedonia [69]. On the other hand, minocycline is also an antibiotic, and antibiotic treatments have been shown to increase the risk for depression and anxiety [70, 71]. Multiple other treatments that target the gut microbiota have also been shown to modulate affective behaviors [72, 73]. Consistent with these findings, we have previously shown a link between SCI-induced intestinal dysbiosis and the development of anxiety-like behavior in rats [10]. Similarly, we presently show that rats with SCI displayed increased anxiety-like behavior, which could be partly alleviated with minocycline treatment. Anxiolytic and anti-depressive effects of minocycline have been previously shown, mainly attributed to the drug’s anti-inflammatory properties [35, 68, 74, 75]. This is particularly relevant in the context of SCI, in which local and systemic inflammation has been associated with increased anxiety- and depressive-like behaviors that may be further exacerbated by gut dysbiosis [10, 38, 76]. Furthermore, stress itself can increase inflammation, induce dysbiosis and anxiety-like behavior [77, 78]. Previously we have shown that a sham SCI surgery can impact the composition of the intestinal microbiota [10], which is why we used uninjured control groups as opposed to sham groups in the present study.

Minocycline’s anti-inflammatory and neuroprotective effects have been well characterized for a variety of diseases/injury conditions; however, little is known about minocycline’s antibiotic effects on the gut microbiota following SCI and how this would influence other outcome measures. Additionally, looking at the microbiota composition provides a measure of the efficacy of minocycline’s dose and route of administration. Here, we show that 7 days of minocycline treatment had a significant effect on the microbiota diversity and composition regardless of injury, proving that the drug was effective. By 28 days post-injury (21 days after the offset of minocycline treatment), the differences between minocycline treated and untreated groups were reduced, and untreated SCI rats began to display an altered microbiota composition (particularly in the Firmicutes/Bacteroidetes ratio, estimated functional gene profile, and at the order, family, and class taxonomic levels). Research in mice also found significantly altered microbiota composition 28 days after a thoracic SCI [9]. Although we did not observe significant differences between SCI and uninjured rats at 5 or 14 days post-injury, it is possible that there was an acute SCI-induced dysbiosis between the day of injury and 5 days post-injury, as previously reported by our laboratory [10].

Although minocycline treatment had a profound effect on the fecal microbiota composition and diversity, a different trend and time course was observed in the systemic inflammatory markers. At 5- and 14-days post-injury, there were minimal differences between groups in the relative change in systemic cytokine/chemokine levels. There was a distinct trend in the plasma analytes such that there was a decrease in the majority of cytokines/chemokines measured relative to baseline in all groups at 5 days post-SCI, including uninjured rats. This may be a result of the stress of surgeries and/or gavaging having a greater acute impact on systemic inflammation than the SCI itself, since stress is known to affect the inflammatory response [79, 80]. Nevertheless, the majority of differences between groups in plasma analytes were observed at 28 days, when untreated SCI rats displayed a reduction in plasma cytokines/chemokines relative to baseline compared to both uninjured groups and SCI + minocycline rats. This finding is somewhat counterintuitive, since increased inflammation is inherent to SCI [81]. Although SCI results in a local inflammatory cascade at the injury site [82], it has been shown that the temporal systemic (i.e. plasma) and local spinal cytokine profiles can be entirely different [83]. One study also found a general SCI-induced downregulation of blood levels of cytokines, which was greater in cervical SCI compared to thoracic SCI [84]. This long-term suppression of cytokines and chemokines may indicate a symptom of SCI-induced immune suppression syndrome. SCI-induced immune suppression is hypothesized to be caused by autonomic dysreflexia triggered by upper thoracic and cervical SCIs [3, 85]. Accordingly, minocycline treatment has been shown to reduce the severity of autonomic dysreflexia after SCI, which may explain how minocycline prevented SCI-induced suppression of inflammatory cytokines/chemokines in the present study. Squair et al. report that, although minocycline treated rats had no observable differences in motor recovery, they had increased preservation of sympathoexcitatory axons and improved cardiovascular control measured 8 weeks following SCI. This chronic setting is when autonomic dysreflexia typically manifests (around 3–6 months after SCI in humans). Similarly, in the present study we did not observe SCI-induced suppression of plasma cytokines until the latest time point measured at 28 days post-SCI. Recent [86] observations from our laboratory corroborate this result and show even more drastic downregulation of blood levels of cytokines/chemokines at 11 weeks after a cervical contusion SCI. Complications due to infection are a leading cause of death following SCI [11], therefore preventing immune suppression may mitigate the risk of infection and thus reduce mortality rates. However, future work would be needed to determine whether the observed SCI-induced reduction of systemic cytokines/chemokines is indeed a symptom of immune suppression, and whether minocycline can prevent SCI-induced immune suppression. Given that the development of immune suppression is dependent on the level of the SCI [3], future work should also consider replicating the present results following different injury levels (for example thoracic and lumbar) and various lesion severities. Furthermore, given the sex differences in immune response [42], this study should be repeated in male rats.

The present results point towards a temporal relationship between minocycline’s effects on the fecal microbiota followed by the reduction in systemic cytokines/chemokines and attenuation of anxiety-like behavior. The gut microbiota plays a critical role in the host immune system, and modulation of the microbiota can have a profound influence on the body’s response to infection and disease [36, 37, 87, 88]. Research in germ free mice (i.e., without any microorganisms) has revealed the vital interplay between the gut microbiota and immune homeostasis [89, 90]. For example, germ free mice have altered macrophage and microglia phenotypes, are neutropenic, and have an impaired innate immune response [91–93]. Germ free mice and mice given antibiotics have also been shown to have a significantly attenuated severity of autoimmune encephalomyelitis via modulation of the peripheral immune response [94, 95]. Furthermore, ongoing research on the gut microbiota strongly suggests a causal link between intestinal dysbiosis and the development of anxiety and depressive-like behaviors [74, 96–98].

## Conclusions

Although our present results are descriptive in nature, they highlight two important concepts. First, although minocycline has direct local anti-inflammatory properties, its impact on the microbiota may also affect the systemic immune and affective consequences of SCI. Second, changes in plasma cytokine/chemokine levels were preceded by minocycline-induced changes in the fecal microbiota composition, suggesting that the microbiota may be involved in the suppression of inflammatory cytokines/chemokines following SCI. In conclusion, our work underscores the importance of the microbiota-immune axis for recovery following SCI, which should be considered when investigating potential therapeutics that may modulate this axis, such as minocycline. The results of the present study are critical for a comprehensive understanding of the full spectrum of minocycline activity beyond the lesion site. This is particularly relevant for the potential clinical application of minocycline to treat acute SCI in humans.

## Supplementary Information

**Additional file 1.** Body weight was monitored at baseline and weekly following SCI. SCI rats lost weight relative to uninjured animals that remained significant until 4 weeks post-injury, particularly in comparison to uninjured + minocycline rats that consistently weighed slightly more than untreated rats. Error bars represent the standard error of the mean. *p<0.05, **p<0.01, ***p<0.001, ****p<0.0001.

**Additional file 2.** Minocycline treatment attenuated spinal cord injury-induced suppression of cytokines/chemokines. Table shows plasma analytes that are significantly different between groups at each time point measured following SCI. P value was calculated using Tukey’s multiple comparison test following a repeated measures two-way ANOVA.

**Additional file 3.** Non-metric multidimensional scaling at the species level shows an effect of minocycline treatment on the overall microbiota composition at 5 and 14 days.

**Additional file 4.** Non-metric multidimensional scaling at the genus level shows an effect of minocycline treatment on the overall microbiota composition at 5 and 14 days.

**Additional file 5.** Non-metric multidimensional scaling at the family level shows an effect of minocycline treatment on the overall microbiota composition at 5 and 14 days. By 28 days, the minocycline effect was reduced, and SCI rats diverged from all other groups in NMDS component 2.

**Additional file 6.** Non-metric multidimensional scaling at the class level shows an effect of minocycline treatment on the overall microbiota composition at 5 and 14 days. By 28 days, the minocycline effect was reduced, and SCI rats diverged from all other groups in NMDS component 2.

**Additional file 7.** Non-metric multidimensional scaling at the order level shows an effect of minocycline treatment on the overall microbiota composition at 5 and 14 days. By 28 days, the minocycline effect was reduced, and SCI rats diverged from all other groups in NMDS component 2.

**Additional file 8.** Top 10% most relative abundant PiCRUST pathways with respect to baseline values.

**Additional file 9.** The number of pathways significantly different between groups (out of the top 10% most abundant PiCRUST pathways). On the day of injury, differences were only observed within minocycline group. At 5 days, minocycline treatment accounted for the majority of differences between groups. By 28 days, the majority of differences were between SCI vs. uninjured and SCI vs. SCI + minocycline groups.

## Data Availability

The dataset supporting the conclusions of this article is available at the Open Data Commons for Spinal Cord Injury (odc-sci.org) (doi:10.34945/F5F30N and doi:10.34945/F5JS3M).

## Abbreviations

DOI: Day of injury
DPI: Days post-injury
EPM: Elevated plus maze
LDB: Light-dark box
NMDS: Non-metric multidimensional scaling
SCI: Spinal cord injury
IL: Interleukin
IP: Interferon gamma-induced protein
GRO/KC: Human growth-regulated oncogene/keratinocyte chemoattractant
TNF: Tumor necrosis factor
G(M)-CSF: Granulocyte (macrophage) colony-stimulating factor
MCP: Monocyte chemoattractant protein-1
MIP: Macrophage inflammatory proteins
RANTES: Regulated upon activation, normal T cell expressed and presumably secreted
VEGF: Vascular endothelial growth factor
