# Supplementary figures and images for: Beyond the lesion site: minocycline augments inflammation and anxiety-like behavior following SCI in rats through action on the gut microbiota
Source: J Neuroinflammation. 2021 Jun 26;18:144. doi: 10.1186/s12974-021-02123-0 (PMC8234629; doi:10.1186/s12974-021-02123-0)

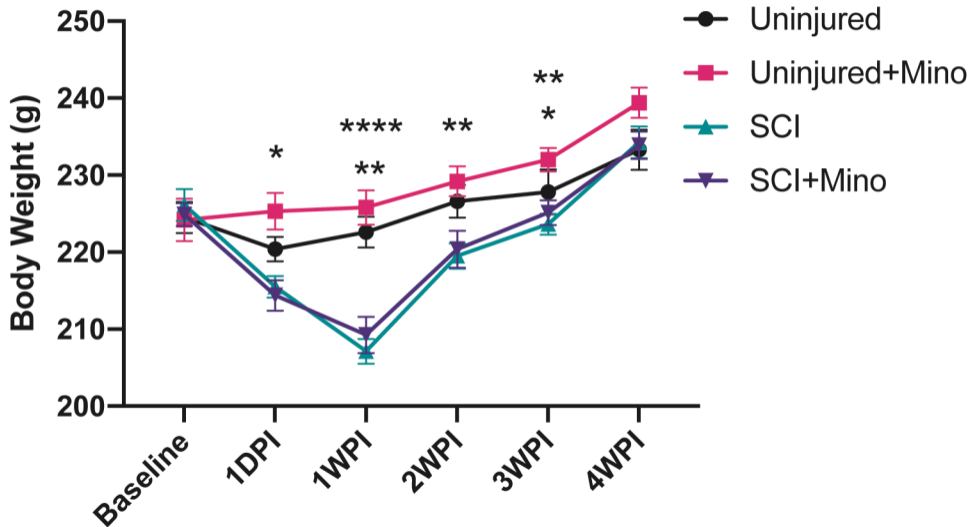

Supplement: Supplementary file 1 — Additional file 1. Body weight was monitored at baseline and weekly following SCI. SCI rats lost weight relative to uninjured animals that remained significant until 4 weeks post-injury, particularly in comparison to uninjured + minocycline rats that consistently weighed slightly more than untreated rats. Error bars represent the standard error of the mean. *p<0.05, **p<0.01, ***p<0.001, ****p<0.0001. [file 12974_2021_2123_MOESM1_ESM.pdf]

## Species

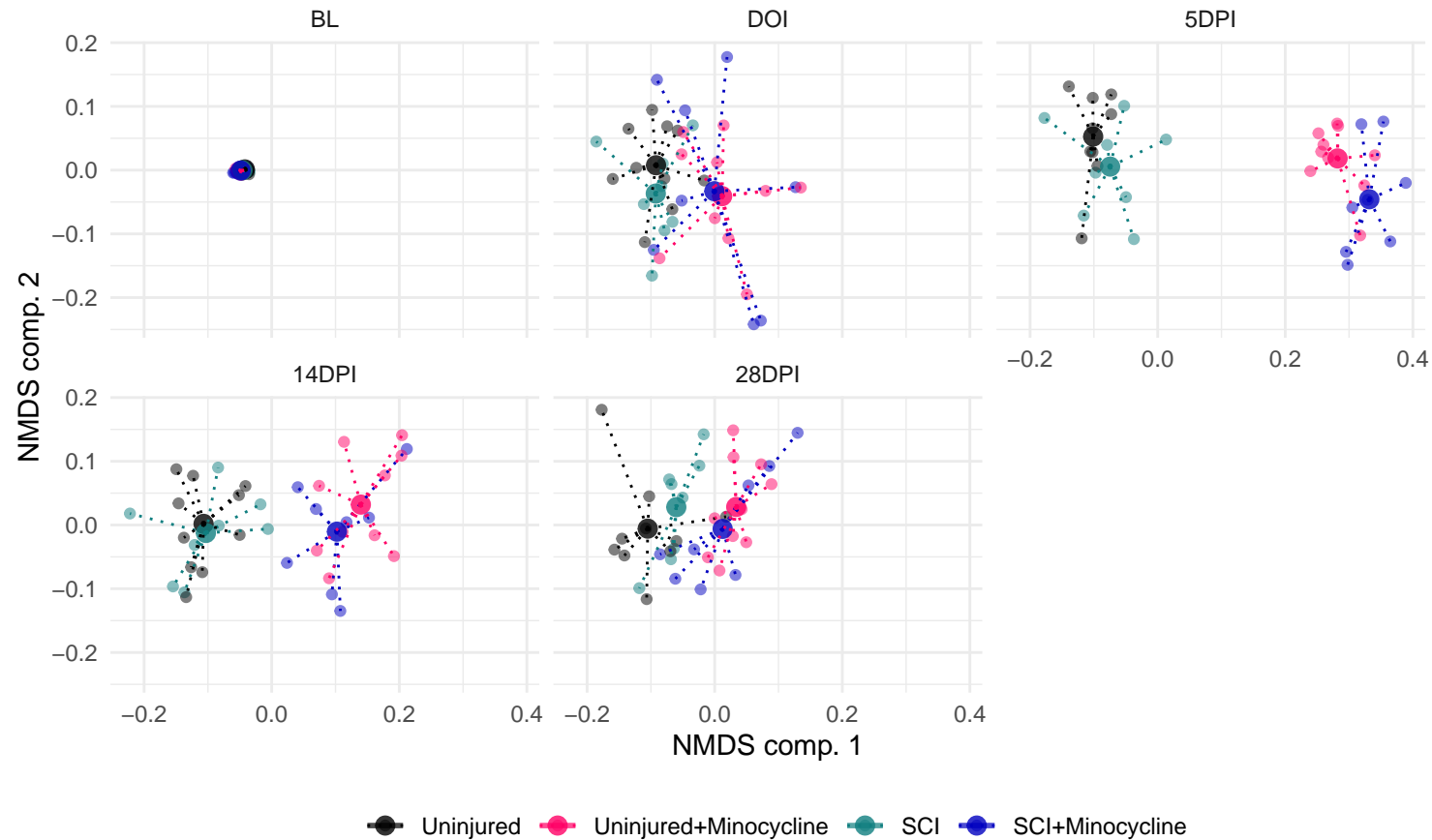

Supplement: Supplementary file 3 — Additional file 3. Non-metric multidimensional scaling at the species level shows an effect of minocycline treatment on the overall microbiota composition at 5 and 14 days. [file 12974_2021_2123_MOESM3_ESM.pdf]

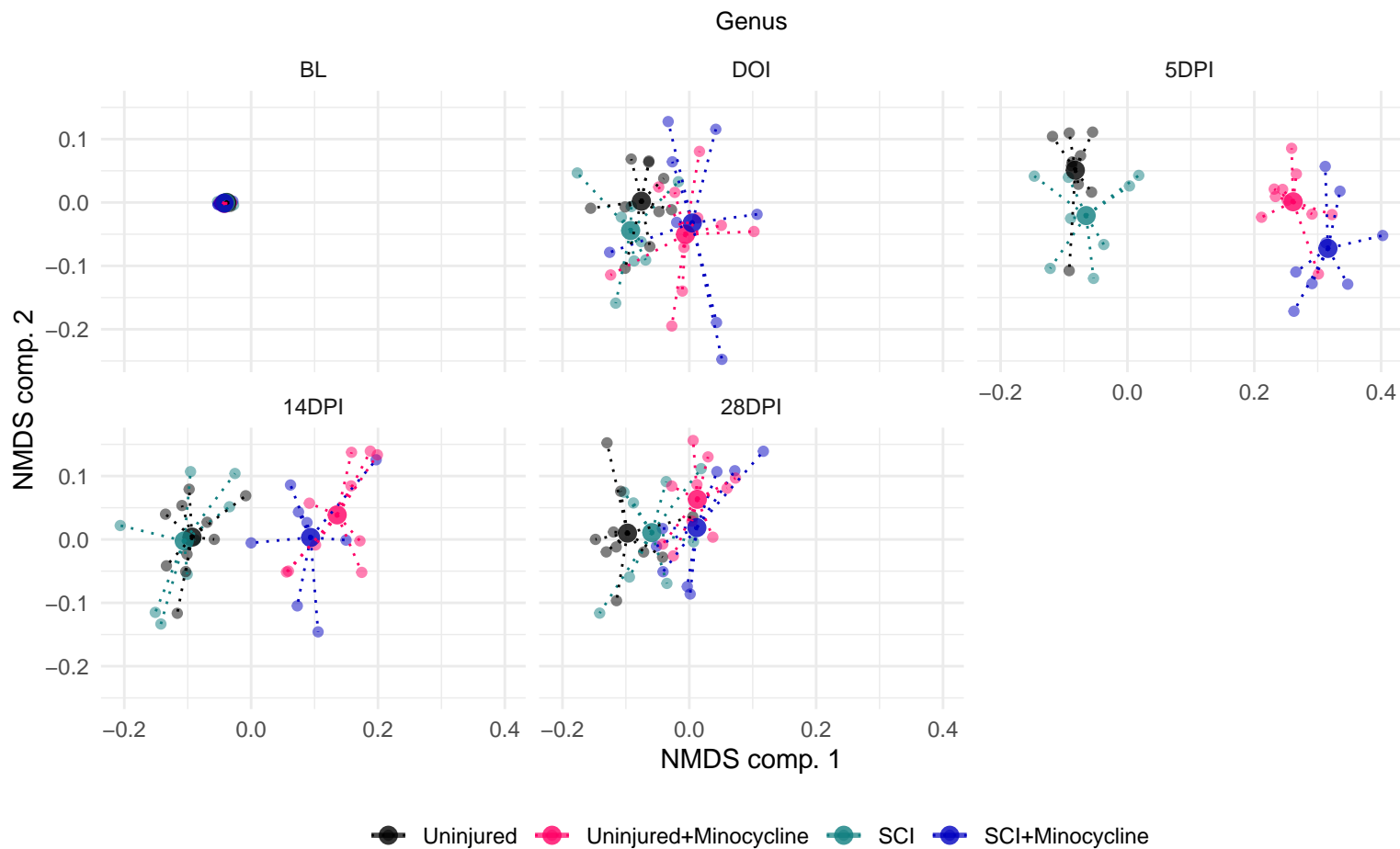

Supplement: Supplementary file 4 — Additional file 4. Non-metric multidimensional scaling at the genus level shows an effect of minocycline treatment on the overall microbiota composition at 5 and 14 days. [file 12974_2021_2123_MOESM4_ESM.pdf]

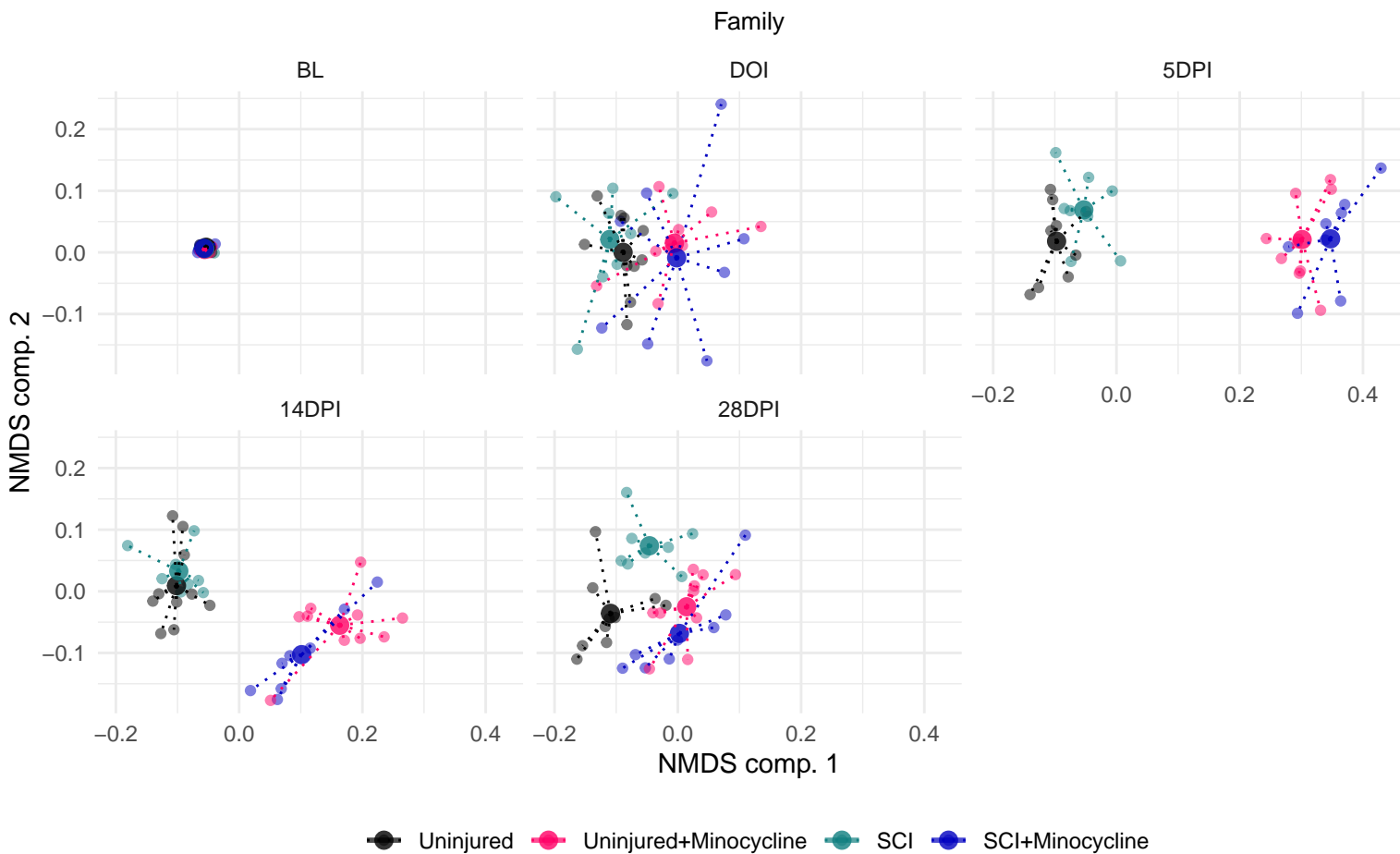

Supplement: Supplementary file 5 — Additional file 5. Non-metric multidimensional scaling at the family level shows an effect of minocycline treatment on the overall microbiota composition at 5 and 14 days. By 28 days, the minocycline effect was reduced, and SCI rats diverged from all other groups in NMDS component 2. [file 12974_2021_2123_MOESM5_ESM.pdf]

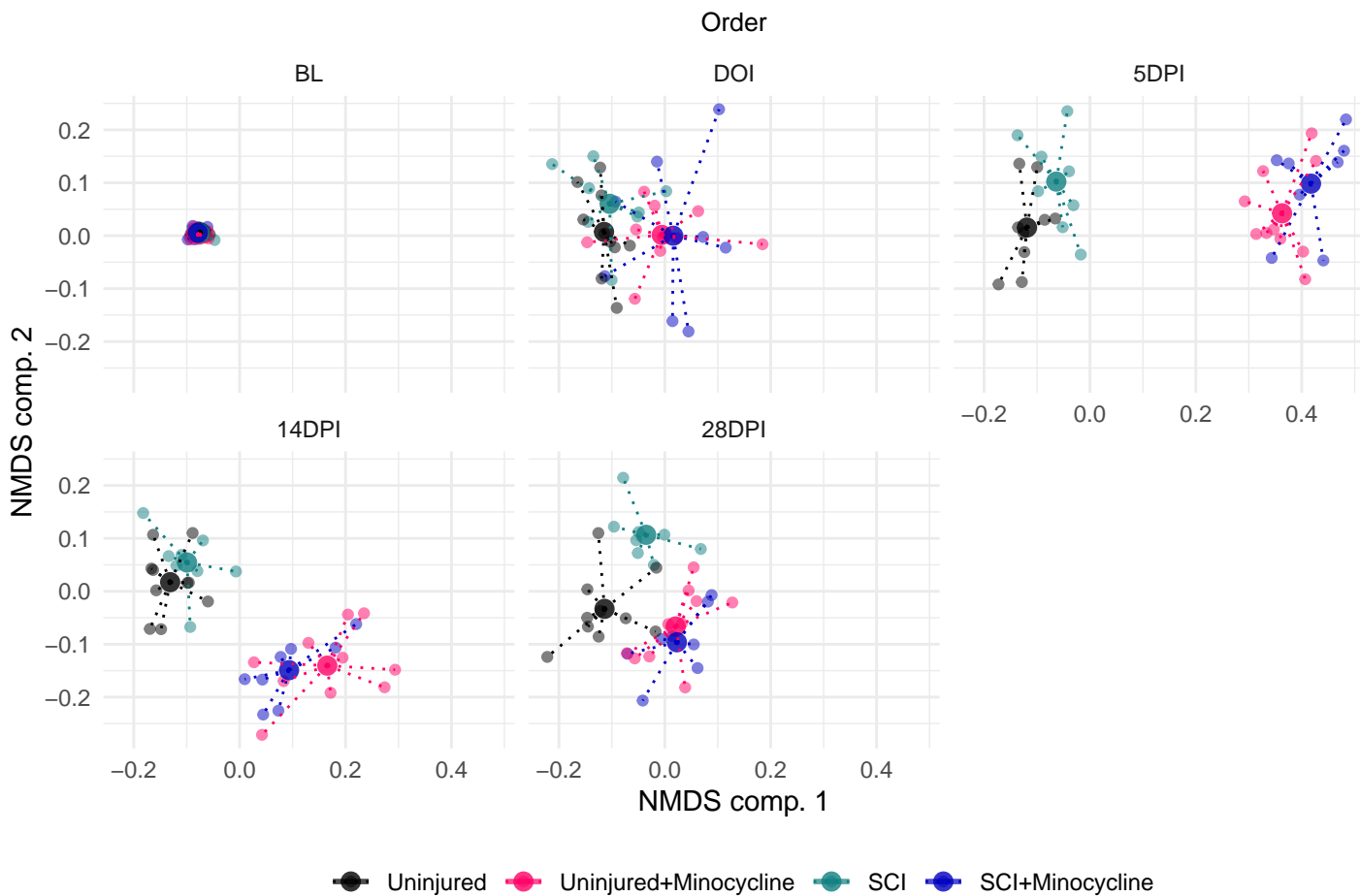

Supplement: Supplementary file 7 — Additional file 7. Non-metric multidimensional scaling at the order level shows an effect of minocycline treatment on the overall microbiota composition at 5 and 14 days. By 28 days, the minocycline effect was reduced, and SCI rats diverged from all other groups in NMDS component 2. [file 12974_2021_2123_MOESM7_ESM.pdf]

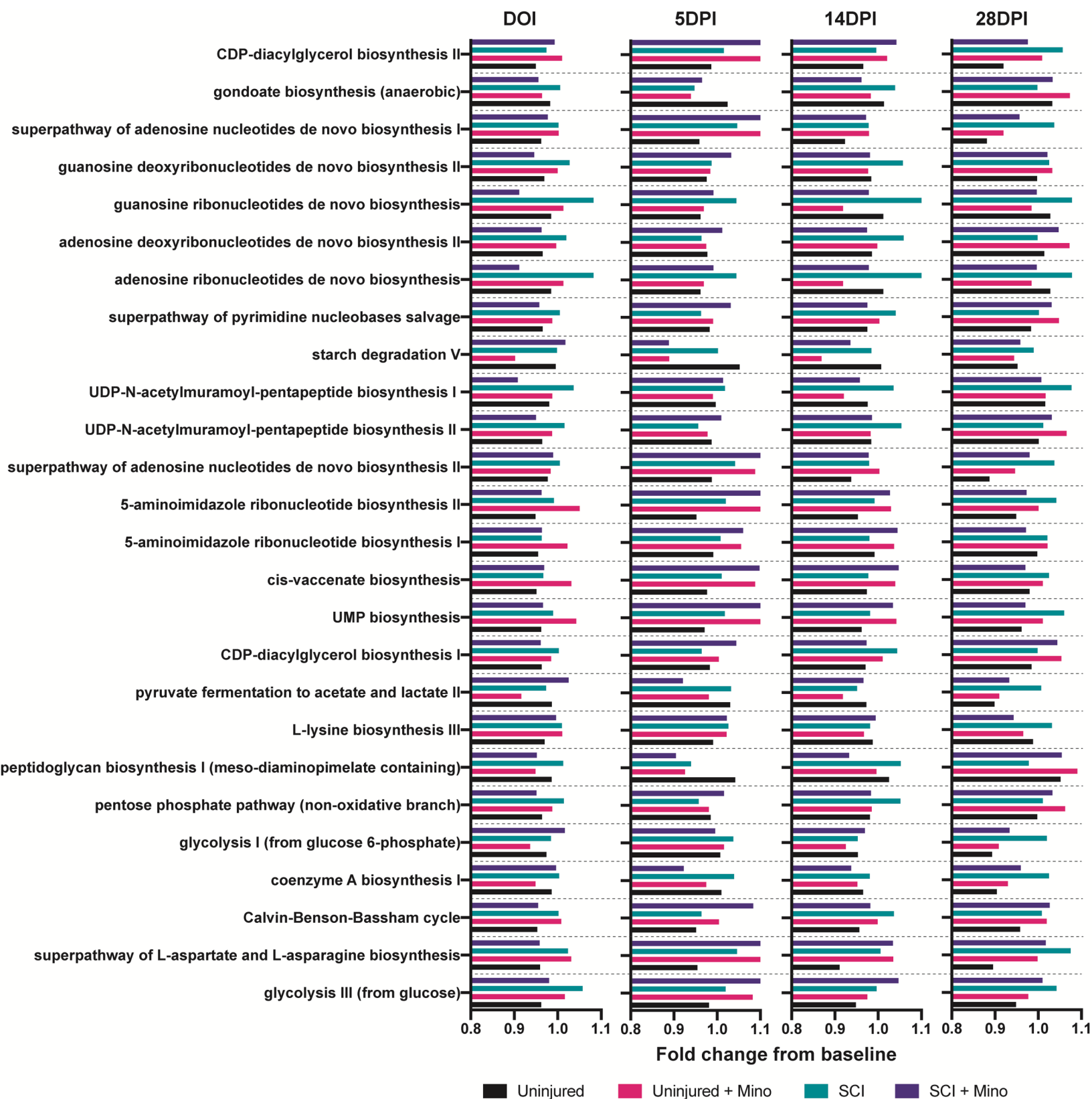

Supplement: Supplementary file 8 — Additional file 8. Top 10% most relative abundant PiCRUST pathways with respect to baseline values. [file 12974_2021_2123_MOESM8_ESM.pdf]

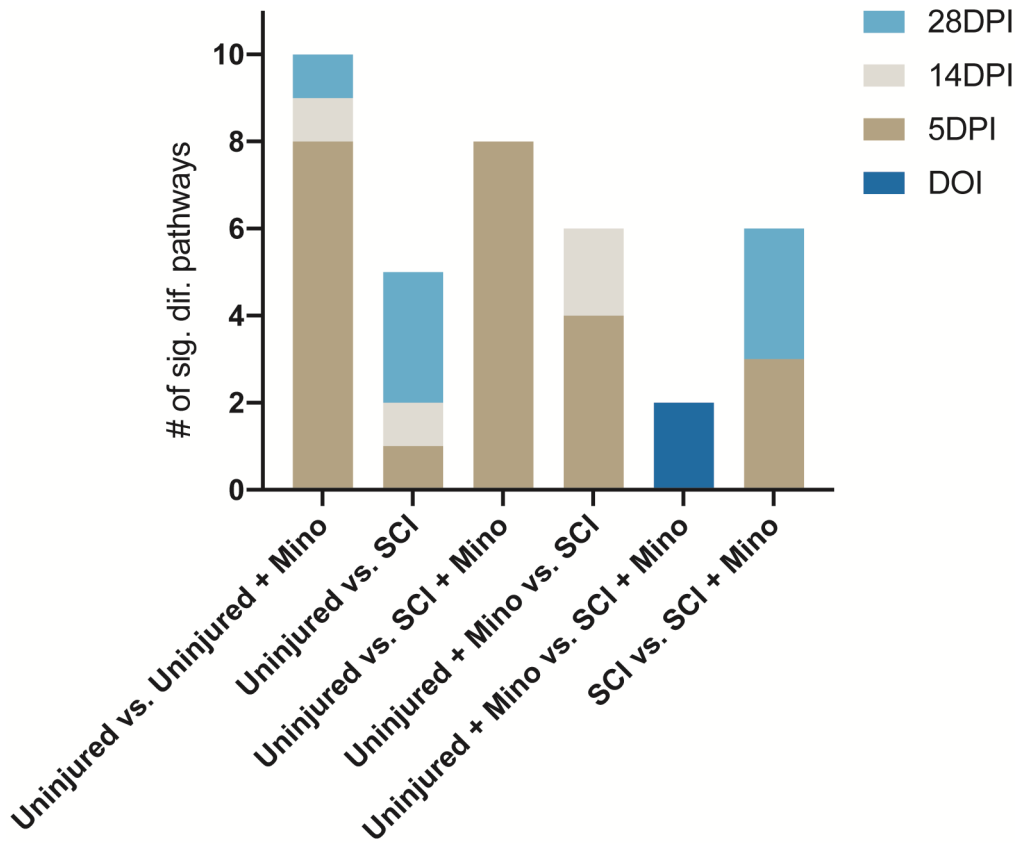

Supplement: Supplementary file 9 — Additional file 9. The number of pathways significantly different between groups (out of the top 10% most abundant PiCRUST pathways). On the day of injury, differences were only observed within minocycline group. At 5 days, minocycline treatment accounted for the majority of differences between groups. By 28 days, the majority of differences were between SCI vs. uninjured and SCI vs. SCI + minocycline groups. [file 12974_2021_2123_MOESM9_ESM.pdf]
