# Supplementary material for: Beyond the lesion site: minocycline augments inflammation and anxiety-like behavior following SCI in rats through action on the gut microbiota
Source: J Neuroinflammation. 2021 Jun 26;18:144. doi: 10.1186/s12974-021-02123-0 (PMC8234629; doi:10.1186/s12974-021-02123-0)
Supplement: Supplementary file 2 — Additional file 2. Minocycline treatment attenuated spinal cord injury-induced suppression of cytokines/chemokines. Table shows plasma analytes that are significantly different between groups at each time point measured following SCI. P value was calculated using Tukey’s multiple comparison test following a repeated measures two-way ANOVA. [file 12974_2021_2123_MOESM2_ESM.pdf]

	5DPI	14DPI	28DPI
Uninjured vs. Uninjured+Mino	Leptin (p = 0.023)	Melatonin (p = 0.049)	
Uninjured vs. SCI		Melatonin (p = 0.041)	IL-12 (p = 0.021)
			Eotaxin (p = 0.041)
			MIP-1a (p = 0.005)
			IL-6 (p = 0.041)
			IL-5 (p = 0.018)
			MCP1 (p = 0.043)
			VEGF (p = 0.044)
			Fractalkine (p = 0.020)
Uninjured vs. SCI+Mino	Leptin (p = 0.026)	Leptin (p = 0.037)	Melatonin (p = 0.026)
		Melatonin (p = 0.012)	
Uninjured+Mino vs. SCI			LIX (p = 0.003)
			IL-17a (p = 0.002)
			IL-12 (p = 0.003)
			GM-CSF (p = 0.003)
			Eotaxin (p = 0.010)
			TNFa (p = 0.005)
			MIP-1a (p = 0.001)
			IL-4 (p = 0.021)
			IL-6 (p = 0.039)
			IL-5 (p = 0.010)
			IL-18 (p = 0.029)
			MCP1 (p = 0.025)
			Fractalkine (p = 0.017)
Uninjured+Mino vs. SCI+Mino			
SCI vs. SCI+Mino			MCP-1 (p = 0.018)
			Eotaxin (p = 0.005)
			GM-CSF (p = 0.015)
			MIP-1a (p = 0.034)
			Fractalkine (p = 0.018)
			IL-6 (p = 0.014)
			TNFa (p = 0.035)
Colour represents which group is significantly increased			
Uninjured	Uninjured + Minocycline	SCI	SCI + Minocycline
