# Supplementary material for: Beyond the lesion site: minocycline augments inflammation and anxiety-like behavior following SCI in rats through action on the gut microbiota
Source: J Neuroinflammation. 2021 Jun 26;18:144. doi: 10.1186/s12974-021-02123-0 (PMC8234629; doi:10.1186/s12974-021-02123-0)
Supplement: Supplementary file 6 — Additional file 6. Non-metric multidimensional scaling at the class level shows an effect of minocycline treatment on the overall microbiota composition at 5 and 14 days. By 28 days, the minocycline effect was reduced, and SCI rats diverged from all other groups in NMDS component 2. [file 12974_2021_2123_MOESM6_ESM.pdf]

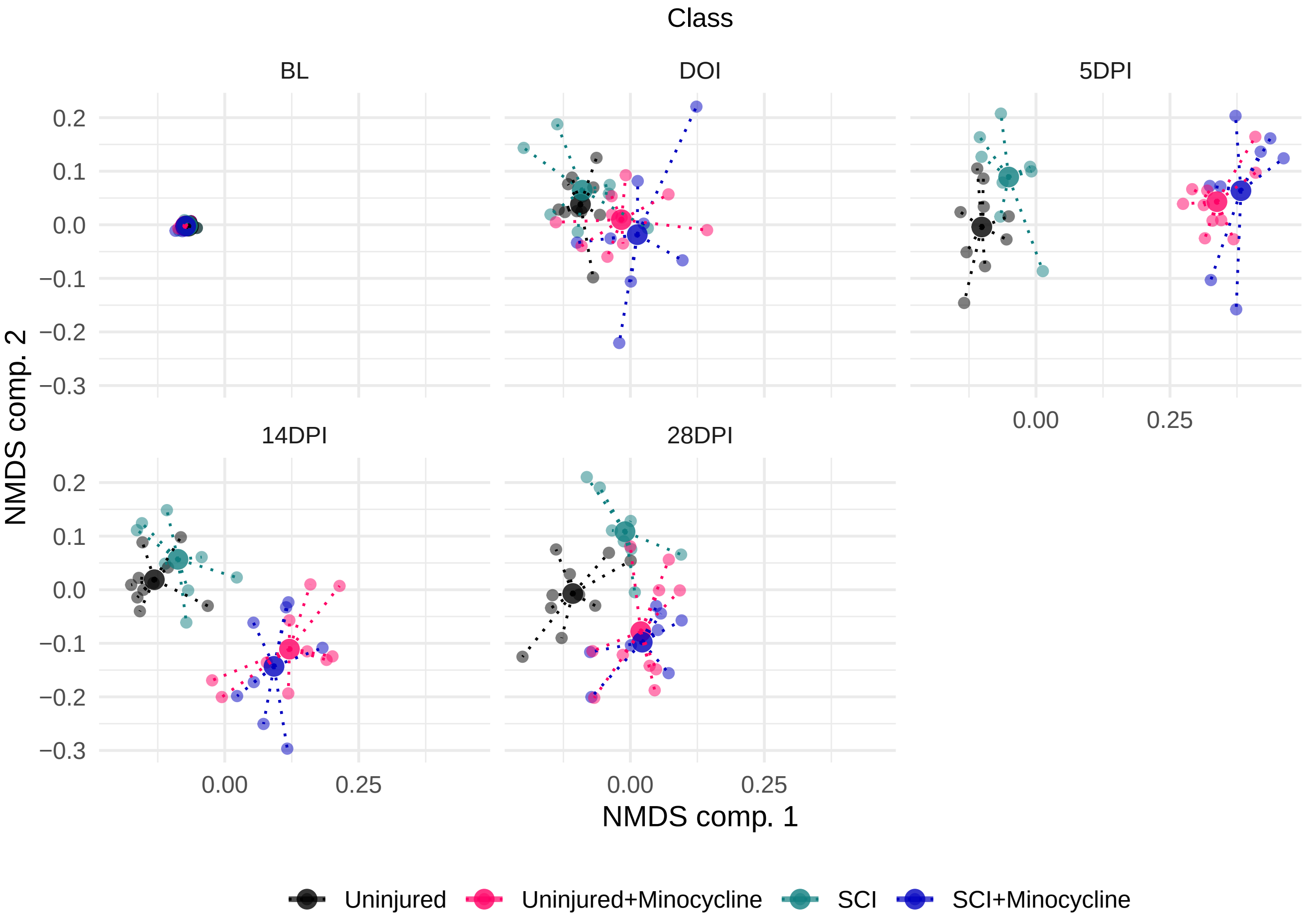

Class
BL
DOI
5DPI
14DPI
28DPI
NMDS comp. 2
NMDS comp. 1
0.2
0.1
0.0
−0.1
−0.2
−0.3
0.00
0.25
Uninjured
Uninjured+Minocycline
SCI
SCI+Minocycline
